# Supplementary material for: From sequence to enzyme mechanism using multi-label machine learning
Source: BMC Bioinformatics. 2014 May 19;15:150. doi: 10.1186/1471-2105-15-150 (PMC4229970; doi:10.1186/1471-2105-15-150)
Supplement: Additional file 2 — Java code of ml2db. Additional file ml2db_code.tar.gz contains the Java source code to run the multi-label machine learning experiments and save the results to database. The code’s Javadoc is included. [file 1471-2105-15-150-S2.zip › additional file 2/ml2db/utils/doc/edu/cornell/lassp/houle/RngPack/class-use/RandomElement.html]

Uses of Class edu.cornell.lassp.houle.RngPack.RandomElement


JavaScript is disabled on your browser.


- Overview
- Package
- Class
- Use
- Tree
- Deprecated
- Index
- Help

- Prev
- Next

- Frames
- No Frames

- All Classes

## Uses of Class edu.cornell.lassp.houle.RngPack.RandomElement

- Packages that use RandomElement

  | Package | Description |
  | --- | --- |
  |  |  |
  | --- | --- |
  | cern.jet.random |  |
  | cern.jet.random.engine |  |
  | edu.cornell.lassp.houle.RngPack |  |
- - ### Uses of RandomElement in cern.jet.random

    Methods in cern.jet.random that return RandomElement

    | Modifier and Type | Method and Description |
    | --- | --- |
    |  |  |
    | --- | --- |
    | `static RandomElement` | AbstractDistribution.`makeDefaultGenerator()` Constructs and returns a new uniform random number generation engine seeded with the current time. |

    Constructors in cern.jet.random with parameters of type RandomElement

    Constructor and Description

    `Pareto(double k,
    double alpha,
    RandomElement randomGenerator)`

    Constructs a Pareto distribution.
  - ### Uses of RandomElement in cern.jet.random.engine

    Subclasses of RandomElement in cern.jet.random.engine

    | Modifier and Type | Class and Description |
    | --- | --- |
    |  |  |
    | --- | --- |
    | `class` | `MersenneTwister` MersenneTwister (MT19937) is one of the strongest uniform pseudo-random number generators known so far; at the same time it is quick. |
    | `class` | `RandomEngine` Abstract base class for uniform pseudo-random number generating engines. |

    Methods in cern.jet.random.engine that return RandomElement

    | Modifier and Type | Method and Description |
    | --- | --- |
    |  |  |
    | --- | --- |
    | `static RandomElement` | RandomEngine.`makeDefault()` Constructs and returns a new uniform random number engine seeded with the current time. |
  - ### Uses of RandomElement in edu.cornell.lassp.houle.RngPack

    Subclasses of RandomElement in edu.cornell.lassp.houle.RngPack

    | Modifier and Type | Class and Description |
    | --- | --- |
    |  |  |
    | --- | --- |
    | `class` | `RandomJava` RandomJava is a class wrapper for the `Math.random()` generator that comes with Java. |
    | `class` | `RandomSeedable` `RandomSeedable` is an abstract class that extends the `RandomElement` class to include the ability to automatically generate a valid `long` seed from the clock. |
    | `class` | `RandomShuffle` RandomShuffle uses one random number generator to shuffle the numbers produced by another to obliterate sequential correlations. |
    | `class` | `Ranecu` Ranecu is an advanced multiplicative linear congruential random number generator with a period of aproximately 10^18^. |
    | `class` | `Ranlux` RANLUX is an advanced pseudo-random number generator based on the RCARRY algorithm proposed in 1991 by Marsaglia and Zaman. |
    | `class` | `Ranmar` RANMAR is a lagged Fibonacci generator proposed by Marsaglia and Zaman and is a good research grade generator. |

    Constructors in edu.cornell.lassp.houle.RngPack with parameters of type RandomElement

    Constructor and Description

    `RandomShuffle(RandomElement ga,
    RandomElement gb,
    int ds)`

- Overview
- Package
- Class
- Use
- Tree
- Deprecated
- Index
- Help

- Prev
- Next

- Frames
- No Frames

- All Classes
